# Ventricular tachycardia without preceding electrocardiogram change after hypertonic mannitol administration: a case report

**DOI:** 10.1186/s40981-018-0191-0

**Published:** 2018-07-23

**Authors:** Akira Gohara, Sumi Okamatsu-Kifuji, Shinjiro Shono, Midoriko Higashi, Ken Yamaura

**Affiliations:** 1 Department of Anesthesiology, Fukuoka University Chikushi Hospital, Chikushino, Japan; 2 Department of Anesthesiology, Fukuoka University Hospital, 7-45-1 Nanakuma, Jonan-ku, Fukuoka, 814-0180 Japan

**Keywords:** Mannitol, Hyperkalemia, Lethal arrhythmia, Ventricular tachycardia

## Abstract

**Background:**

Mannitol is widely used during neurosurgery, but it has a serious complication including lethal arrhythmia due to mannitol-induced hyperkalemia.

**Case presentation:**

We report on a 62-year-old man scheduled for the clipping of an unruptured cerebral artery aneurysm. During surgery, approximately 20 min after the end of 200-mL 20% hypertonic mannitol administration, ventricular tachycardia (VT) occurred without preceding electrocardiogram (ECG) change, such as peaked T waves, and VT was recovered to sinus rhythm after chest compression. A potassium concentration after recovery from VT was 6.4 mEq/L, which was normalized by the administration of calcium gluconate, furosemide, and insulin with glucose.

**Conclusions:**

Physicians must be aware that VT without preceding ECG change can occur after hypertonic mannitol administration.

## Background

Mannitol is used to reduce the intracranial pressure during neurosurgery. A serious complication of mannitol is mannitol-induced hyperkalemia, which causes electrocardiogram (ECG) change (peaked T waves and wide QRS) and subsequent lethal arrhythmia [1–4]. We report a case of ventricular tachycardia (VT) without preceding ECG change after mannitol administration during cerebral artery clipping. We have obtained written consent from the patient to publish this report.

## Case presentation

**Fig. 1:**
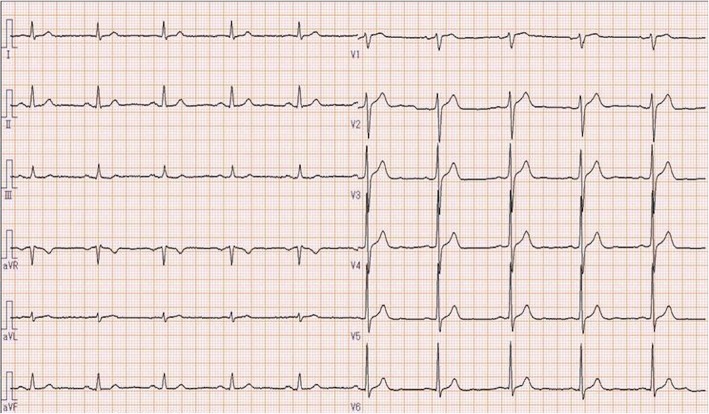
The patient’s 12-lead electrocardiography (ECG) before surgery

A 62-year-old, 173 cm, and 64 kg man was scheduled for the clipping of an unruptured anterior communicating cerebral artery aneurysm via craniotomy. He had previously received Telmisartan and sodium (Na) valproate for hypertension and symptomatic epilepsy after cranial hematoma due to right occipital lobe bleeding under the cortex. Preoperative 12-lead ECG (Fig. 1) and routine laboratory tests, including serum Na (141 mEq/L) and potassium (K) (4.1 mEq/L), were conducted.

Upon the arrival of the patient in the operating room, his hemodynamic state was stable. Anesthesia was induced with propofol (100 mg) and remifentanil (0.5 μg/kg/min), and via intubation with rocuronium (50 mg), and maintained with sevoflurane (1.2–1.5%) and remifentanil (0.05–0.2 μg/kg/min). At 25 min after surgical incision, 200 mL of 20% mannitol (40 g) was intravenously administered over 60 min.

**Fig. 2:**
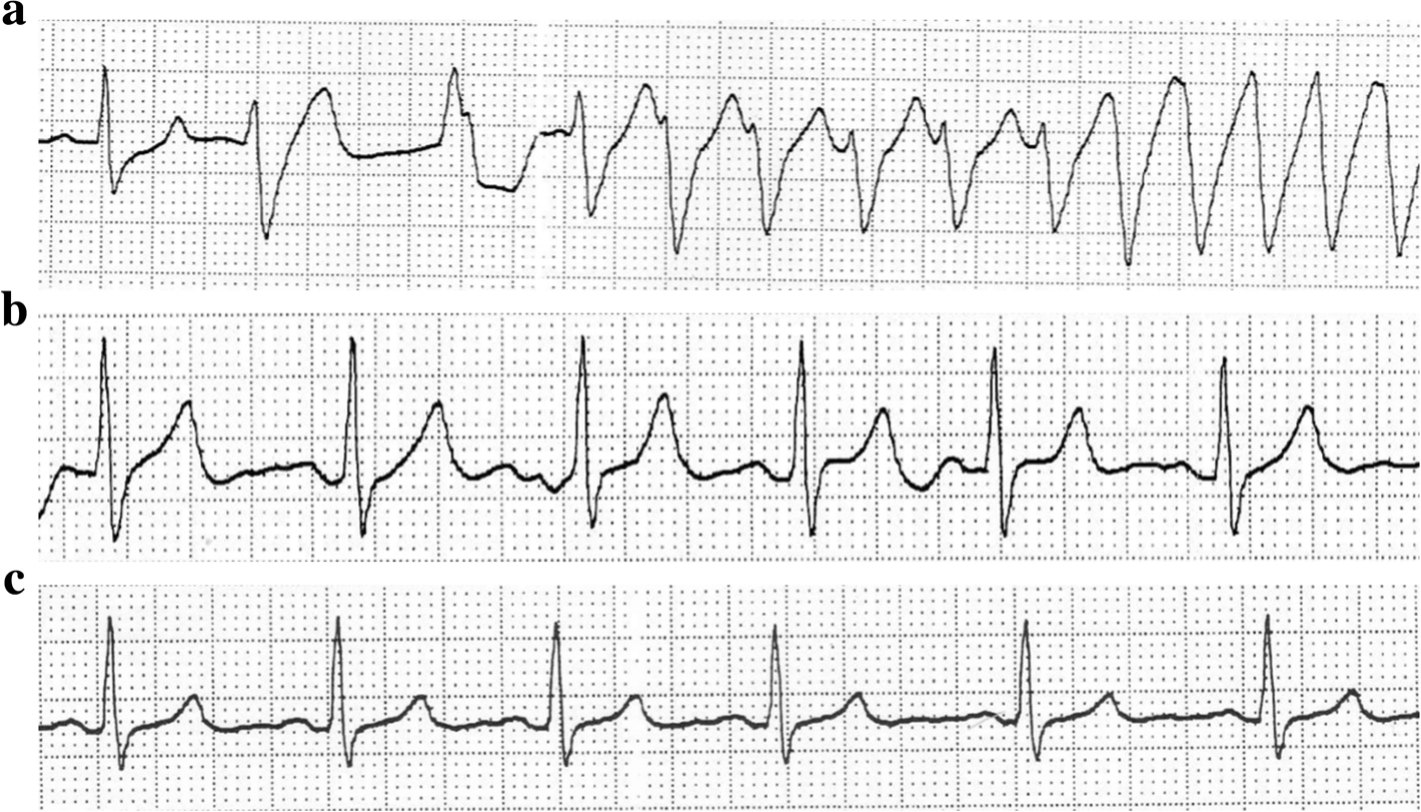
The patient’s ECG (II lead) during surgery. **a** VT developed approximately 20 min after the end of mannitol administration. **b** ECG shows peaked T waves after the recovery of sinus rhythm. **c** The peaked T waves disappeared after 2 min

Approximately 20 min after the end of mannitol administration, VT occurred (Fig. 2a). At that time, in the surgical field, the arachnoid membrane of the Silvius cleft was separated. Blood pressure decreased to < 40 mmHg. We immediately started chest compression, and VT recovered to sinus rhythm at a rate of 75 beats/min approximately after 20 s after chest compressions began. After the recovery of sinus rhythm, ECG showed peaked T waves (Fig. 2b), which disappeared after 2 min (Fig. 2c). Arterial blood gas analysis showed pH 7.43, an arterial carbon dioxide tension (PaCO_2_) of 45 mmHg, an arterial oxygen tension (PaO_2_) of 375 mmHg at an inhaled oxygen concentration of 100%, a base excess of 5.0 mmol/L, and K concentration of 6.4 mEq/L. Body temperature was 36.3 °C. Calcium gluconate (10 mL, 8.5%), furosemide (20 mg), and insulin with glucose (10 U, 25 g) were administered. After 60 min, plasma K concentration was 3.2 mEq/L. The clipping of the cerebral artery was completed without any further cardiac instability. At the end of surgery, plasma K concentration was 3.5 mEq/L and there were no abnormalities due to 12-lead ECG and laboratory tests. The patient was extubated after confirming normal examination data. Surgical time was 4 h and 5 min, estimated blood loss during operation was 225 mL, and 800 mL of 1% glucose-added acetate Ringer solution (Na, 140 mEq/L; K, 4 mEq/L) and 500 mL of normal saline were administered. Urine output was 2250 mL. The patient did not have any further postoperative event.

## Discussion

In this case, VT occurred without any preceding ECG change despite hyperkalemia due to mannitol. Lethal arrhythmia often occurs during neurosurgery. In general, there have been cases during surgical operations near the brainstem with an increased sympathetic nerve system due to the rupture of a cerebral aneurysm and with hemorrhage shock during craniotomy. There are a few reports of fatal arrhythmia, VT, and/or ventricular fibrillation due to mannitol, which mainly is due to hyperkalemia [3–5].

Mannitol is widely used to reduce the intracranial pressure due to an increase in plasma osmolality. However, it has several side effects, including osmotic nephrosis and electrolyte imbalance [6]. The rapid infusion of mannitol can cause hyponatremia or hyperkalemia. Hyponatremia is mainly due to a transient dilution. During mannitol-induced hyperkalemia, intracellular K is thought to be released into the extracellular space due to an acute increase in plasma osmolality, rhabdomyolysis, hemolysis, and acidosis [1, 7]. In this case, preoperative renal function was normal and there were no findings of acidosis, rhabdomyolysis, or hemolysis. The frequency of mannitol-induced hyperkalemia is 7.7%, and the total dose of mannitol as well as its rate of infusion and decreased renal function may have a role in the development of mannitol-induced hyperkalemia [4]. We used a total dose of 40 g mannitol, which was within the recommended dose (0.25–1 g/kg) [8], and the infusion rate was 60 min, which was not rapid.

In most cases, the ECG change—elevated T waves—was the primary finding. All cases of lethal arrhythmia reported thus far have been preceded by ECG change, including peaked T waves and a wide QRS complex [3–5]. However, no preceding ECG change was observed in our case, and transient peaked T waves appeared after recovery to sinus rhythm from VT. With regard to ECG changes in hyperkalemia, such as elevated T wave (tentative T wave), P wave decrease, prolonged PQ interval due to an increase in serum K value, QRS width extension, P wave disappearance and its further increase that leads to an unclear distinction between QRS and T waves, and heartbeats becoming standstill [9]. However, ECG without any finding consistent with hyperkalemia is noted in > 50% of patients with K > 6.5 mEq/L, and arrhythmia and cardiac arrest have been reported in patients with hyperkalemia without a preceding peak T wave [10, 11]. It is not clear why VT occurred in this case, but in addition to transient potassium elevation due to potassium permeability to cardiac membrane by myocardial ischemia [12] and mannitol, craniotomy itself may have influenced it.

Therefore, physicians should be aware that lethal arrhythmia can occur due to mannitol-induced hyperkalemia.

## Conclusions

We present a case of VT without preceding ECG change after mannitol administration. Hyperkalemia should be confirmed regardless of the presence or absence of ECG abnormalities.

## Abbreviations

ECG: Electrocardiogram
VT: Ventricular tachycardia
